# Identification of a Recurrence Gene Signature for Ovarian Cancer Prognosis by Integrating Single-Cell RNA Sequencing and Bulk Expression Datasets

**DOI:** 10.3389/fgene.2022.823082

**Published:** 2022-06-08

**Authors:** Yongjian Zhang, Wei Huang, Dejia Chen, Yue Zhao, Fusheng Sun, Zhiqiang Wang, Ge Lou

**Affiliations:** Department of Gynecology Oncology, Harbin Medical University Cancer Hospital, Harbin, China

**Keywords:** ovarian cancer, single-cell RNA-seq, prognostic model, immune subtypes, recurrence

## Abstract

Ovarian cancer is one of the most common gynecological malignancies in women, with a poor prognosis and high mortality. With the expansion of single-cell RNA sequencing technologies, the inner biological mechanism involved in tumor recurrence should be explored at the single-cell level, and novel prognostic signatures derived from recurrence events were urgently identified. In this study, we identified recurrence-related genes for ovarian cancer by integrating two Gene Expression Omnibus datasets, including an ovarian cancer single-cell RNA sequencing dataset (GSE146026) and a bulk expression dataset (GSE44104). Based on these recurrence genes, we further utilized the merged expression dataset containing a total of 524 ovarian cancer samples to identify prognostic signatures and constructed a 13-gene risk model, named RMGS (recurrence marker gene signature). Based on the RMGS score, the samples were stratified into high-risk and low-risk groups, and these two groups displayed significant survival difference in two independent validation cohorts including The Cancer Genome Atlas (TCGA). Also, the RMGS score remained significantly independent in multivariate analysis after adjusting for clinical factors, including the tumor grade and stage. Furthermore, there existed close associations between the RMGS score and immune characterizations, including checkpoint inhibition, EMT signature, and T-cell infiltration. Finally, the associations between RMGS scores and molecular subtypes revealed that samples with mesenchymal subtypes displayed higher RMGS scores. In the meanwhile, the genomics characterization from these two risk groups was also identified. In conclusion, the recurrence-related RMGS model we identified could provide a new understanding of ovarian cancer prognosis at the single-cell level and offer a reference for therapy decisions for patient treatment.

## Introduction

Ovarian cancer is one of the most common gynecological malignancies in women, with a poor prognosis and high mortality (Momenimovahed et al., 2019). Globally, approximately 300, 000 new cases and 185, 000 deaths are reported each year (Bray et al., 2018). Since the early stage is mostly asymptomatic, ovarian cancer is usually diagnosed at an advanced stage, which accounts for a 5-year survival rate of 30% (Croft et al., 2021). Surgical resection and systemic adjuvant chemotherapy remain the mainstay of ovarian cancer treatment; however, chemo-resistance develops in up to 75% of patients. In addition, most patients present with chronic recurrences, and approximately 75% of those patients are incurable among women with advanced OC (Lheureux et al., 2019). Moreover, the patients with recurrence tumors usually displayed poor prognosis. Currently, early detection strategies, treatments, and monitoring to improve survival and quality of life are also inadequate (Alexandrova et al., 2020). Therefore, there is an urgent need to explore the biological mechanism of ovarian cancer recurrence and identify the novel signature model as an effective predictor of ovarian cancer patients.

Recently, previous studies have identified kinds of single genes and multiple genes as the ovarian cancer prognosis signatures. For example, it was revealed that forkhead box M1 (FOXM1) regulated cell proliferation, invasion and metastasis, chemo-resistance, and finally promoted poor prognosis in ovarian cancer patients (Liu et al., 2021). Mitochondrial ribosomal protein L15 (MRPL15) is observed highly expressed in epithelial ovarian cancer and is also associated with poor patient overall survival (Xu et al., 2021). The high expression of DOT1-like histone lysine methyltransferase (DOT1L) could promote ovarian cancer cell growth by regulating apoptotic and metabolic pathways, affecting patient prognosis (Chava et al., 2021). In a clinical cohort, flap structure-specific endonuclease 1 (FEN1) is identified as a key molecule in DNA repair, and FEN1 overexpression is associated with poor survival after platinum chemotherapy of ovarian cancer patients (Mesquita et al., 2021). Furthermore, multiple gene-based signatures were also identified for predicting patient survival. Zhang et al. (2021)constructed a glycolysis-related gene signature for ovarian cancer survival prediction, which included nine genes. In another study, the differentially expressed genes between primary tumor and metastatic tumor samples were identified, and weighed gene correlation network analysis and module analysis were performed to further identify metastasis-specific genes for ovarian cancer (Gu and Zhang, 2021). However, most of these studies focused on the bulk tissue expression profiles for identifying prognostic or metastatic signatures, with the limitation of tumor purity or immune cell infiltration analysis.

Transcriptome studies of single-cell RNA sequencing for ovarian cancer have revealed the cell-level heterogeneity of the tumor microenvironment, improving our understanding of the biological mechanisms of cancer initiation, recurrence, and drug resistance (Hoffman et al., 2020; Izar et al., 2020). Utilization of a single-cell RNA sequencing dataset to identify potential drivers and therapeutic targets for ovarian cancer can help predict patient prognosis or explore the recurrence mechanism (Hao et al., 2021). Wang et al., 2022performed the integrated analysis of bulk RNA-seq samples and single-cell RNA sequencing dataset for identifying survival-related markers, and the stem cell population involved in ovarian cancer cells and potential treatment recommendations were explored. To characterize tumor cell heterogeneity and the infiltration of M2 tumor-associated macrophages (TAMs) in the ovarian cancer TME, Liu et al., 2022performed the computational analysis by integrating single-cell RNA sequencing with bulk RNA-seq datasets, and four M2 TAM-associated genes correlated with survival were identified. Moreover, the biological mechanism involved in ovarian cancer recurrence at the single-cell level, and the associations between recurrence cell markers and patient prognosis were urgently explored.

In this study, we systematically explored the functional difference of ovarian cancer recurrence by integrating one single-cell RNA sequencing dataset and one bulk expression dataset from the Gene Expression Omnibus (GEO) database. Based on the recurrence-related genes and merged expression profiles with patient survival derived from seven GEO datasets, we constructed a novel risk model, named RMGS (recurrence marker gene signature), using lasso cox analysis. Based on the RMGS score, the ovarian cancer samples could be divided into two risk groups in an independent cohort, including The Cancer Genome Atlas (TCGA) dataset and another GEO dataset. The biological functions involved in two RMGS groups were revealed, and the difference in the immune checkpoint expression was also observed. Finally, we explored the associations between the RMGS score and ovarian cancer molecular subtypes and genomics mutation features.

## Materials and Methods

### Data Source and Acquisition

We searched available single-cell RNA sequencing datasets with ovarian cancer recurrence information from the GEO database. Also, one dataset GSE146026 containing 12 ovarian cancer samples from two technologies (six from 10X and six from smartseq2) was obtained in the form of RSEM normalized counts. All these 12 samples belonged to the high-grade serous carcinoma. The following analyses were performed for single-cell RNA sequencing datasets with two platforms respectively. Also, we further obtained one bulk expression dataset (GSE44104) with recurrent information from the GEO database, which contained a total of 60 ovarian cancer samples (Wu et al., 2014). For prognostic model construction, a total of seven ovarian cancer expression datasets with survival information were obtained from the GEO database, including GSE14764 (n = 80) (Denkert et al., 2009), GSE19829 (n = 28) (Konstantinopoulos et al., 2010), GSE23554 (n = 28) (Marchion et al., 2011), GSE26193 (n = 107) (Gentric et al., 2019), GSE26712 (n = 185) (Bonome et al., 2008), GSE30161 (n = 58) (Ferriss et al., 2012), and GSE63885 (n = 101) (Lisowska et al., 2016). The platform of all these datasets was Affymetrix Human Genome Array (GPL96, U133A and GPL570, and U133 Plus 2.0). The samples without available survival time or corresponding survival time of less than 30 days were removed, and a total of 524 samples were considered as the training samples. To validate the ability of the prognostic model, the individual patient survival information and mRNA expression dataset from the TCGA database were downloaded. The samples with a survival time of less than 30 days were removed, and a total of 370 ovarian cancer samples with high-grade serous type were obtained. Another GEO dataset (GSE140082, n = 380) (Kommoss et al., 2017) whose platform was Illumina HumanHT-12 beadchip was also obtained for prognostic validation analysis.

### Identification of Recurrence-Related Marker Genes

The ovarian cancer single-cell RNA sequencing dataset was analyzed by using the “Seurat” package for two technologies, respectively (Aran et al., 2019). First, we removed the cells with more than 5% of mitochondrial genes. Similarly, the cells with number of genes mapped less than 200 and samples with cell counts less than five were moved. We then performed principal component analysis (PCA) using the most 1500 variable genes in order to visualize transcriptional variability over the complete single-cell RNA sequencing dataset. T-distributed Stochastic Neighbor Embedding (t-SNE) was used for further dimensional reduction of the significant principal components (Hoffman et al., 2020). Second, we annotated the cell types for two datasets using the “SingleR” package, and the transcriptome profiles of malignant epithelial cells were obtained for the following analysis. Finally, we identified the recurrence-related genes involved in epithelial cells. The genes that exhibited a |log2 (fold change)| > 1 and adjusted *p*-value < 0.05 of the epithelial cells between recurrence samples and primary samples were identified from the single-cell dataset. For the bulk expression dataset, the differentially expressed analysis was performed between recurrence and primary samples using the “limma” package. Also, the genes that exhibited a |log2 (fold change)| > 1 and *p*-value < 0.05 were considered as the recurrence genes from the bulk dataset.

### Construction of the Recurrence Marker Gene Risk Model (RMGS)

Based on the recurrence-related genes obtained from single-cell RNA sequencing and bulk dataset, we further constructed a prognostic model using seven expression profiles from the GEO database. First, we constructed a merged expression matrix based on these seven expression datasets which contained available survival information. The combat algorithm was utilized for removing the batch effect of different datasets. Then, for the common recurrence genes between single-cell RNA sequencing and bulk expression datasets, the lasso cox regression model was performed by using the “glmnet” package, with the optimal lambda value determined by 10-fold cross-validation (Goeman, 2010). Also, these resulting genes were further included in the recurrence marker gene signature (RMGS). The RMGS contained 11 genes, and the risk formula was provided as follows:

$$\text{EMGS}=\overset{11}{\underset{\text{i}=1}{\sum }}{\text{Coef}}_{\text{i}}\times {\text{Exp}}_{\text{i}},$$

where Coef_i_ is the lasso coefficient value for the *i*th recurrence markers, and Exp_i_ is the expression value.

### Statistical Analysis

According to the RMGS score, all ovarian cancer samples from the training and validation set were divided into two groups. Then, the Kaplan–Meier (KM) curve and survival *p*-value calculated by the log-rank test were performed using the “survminer” package. The Lasso cox regression model analysis was performed for the RMGS model construction by using the “glmnet” package. Univariate and multivariate Cox regression model analyses were performed for evaluating RMGS performance by using “survival” and “survminer” packages. Wilcoxon test was used to determine statistical differences of categorical variables between two RMGS groups. The Pearson correlation coefficient with |r|>0.3 and *p*-value < 0.05 were defined as a significantly correlated association. All figure construction in this study was conducted by R package software (version 4.0.3).

## Results

### Identification and Analysis of Marker Genes Associated With Recurrence

**FIGURE 1:**
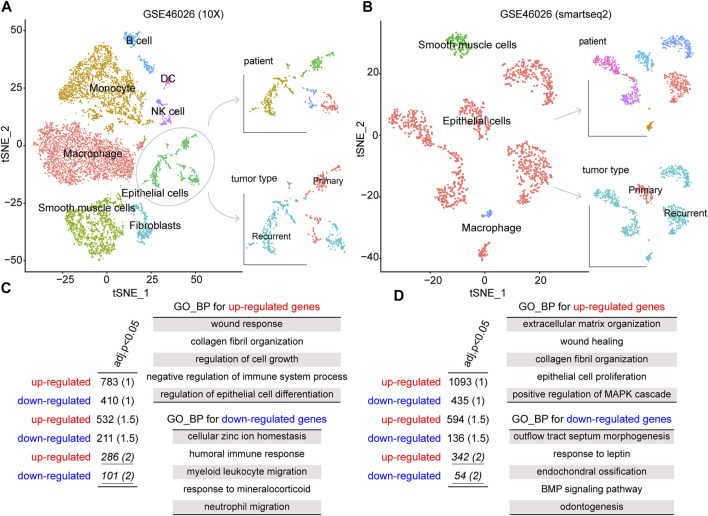
tSNE plot of all cells collected by GSE146026, color by cell type, patients, and tumor type in the **(A)** 10X platform and **(B)** smartseq2 platform. The gene ontology (GO)–biological process (BP) terms were identified from dysregulated genes for **(C)** 10X platform and **(D)** smartseq2 datasets.

To explore the biological mechanism involved in ovarian cancer recurrence at the single-cell level, we obtained a single-cell RNA sequencing dataset, GSE146026, for identifying recurrence-related genes. Also, the two expression matrices obtained from 10X and smartseq2 technologies were, respectively, analyzed (see Materials and Methods). First, we reduced the dimensionality of the data by PCA by using the 1500 variable genes. We then assigned cell-type identities by cross-referencing differentially expressed genes in each cluster with previously reported cell-type-specific marker genes using the Seurat package. A total of eight cell clusters were identified from the 10x dataset, including macrophages, monocytes, smooth muscle cells, epithelial cells, fibroblasts, B cells, NK cells, and DC (Figure 1A). Moreover, three cell clusters were identified from the smartseq2 dataset, including macrophages, epithelial cells, and smooth muscle cells (Figure 1B). As a result, the epithelial cells displayed heterogeneity among ovarian cancer patients for both 10x and smartseq2 datasets. In the meanwhile, the difference between epithelial cells from primary and recurrent samples was also apparent.

To identify the gene signatures expressed by malignant cells between recurrence and primary samples, we performed the differential expression analysis for epithelial cells between these two groups. By a strict cutoff, we identified 286 upregulated and 101 downregulated genes from GSE46026 (10X) and 342 upregulated and 54 downregulated genes from GSE46026 (smartseq2). Then, Gene Ontology (GO) Biological Process (BP) enrichment analysis was performed to explore the biological function of these recurrence genes using the clusterProfiler package (Yu et al., 2012). As shown in Figure 1C,D, some common functions such as wound response and epithelial cell proliferation were both identified by upregulated genes. Also, the immune response function was identified by the downregulated genes from GSE46026 (10X), and the BMP signaling pathway was identified by the downregulated genes from GSE46026 (smartseq2). Therefore, the union of these two dysregulated genes was regarded as the recurrence-related genes from the single-cell dataset. Furthermore, the detailed function enrichment results for GSE46026 (10X) and GSE46026 (smartseq2) are shown in Supplementary Tables S1, S2. Meanwhile, we identified the recurrence-related genes from the bulk expression dataset, GSE44104 (see Materials and Methods). Also, 133 upregulated and 100 downregulated genes were identified, and the corresponding biological functions were also explored (see Supplementary Figure S1 and Supplementary Table S3). Finally, the 39 common genes shared by single-cell and bulk expression datasets were regarded as the recurrence genes.

### Construction of the Recurrence Marker Gene Signature (RMGS) for Ovarian Cancer Prognosis

Next, we utilized the gene expression data on ovarian cancer samples with survival data from a total of seven datasets to further identify prognostic signatures based on recurrence genes (see Materials and Methods). As a result, 11 of the 39 recurrence genes were identified from the 524 ovarian cancer samples as the training samples, which included *BIRC3*, *CDH2*, *CDH6*, *DDIT4*, *GAS1*, *IFIT1*, *IGF2*, *ISLR*, *MUC16*, *RSAD2*, and *DIRAS3*. Considering the lasso coefficient of these genes, we further constructed a prognostic model named RMGS, recurrence marker gene signature. In detail, the formula for calculating the RMGS score was displayed as follows: RMGS score = BIRC3 expression X (−0.005) + CDH2 expression X (−0.044) + CDH6 expression X (0.026) + DDIT4 expression X (0.046) + GAS1 expression X (0.009) + IFIT1 expression X (0.013) + IGF2 expression X (0.042) + ISLR expression X (0.068) + MUC16 expression X (−0.048) + RSAD2 expression X (0.008) + DIRAS3 expression X (−0.041).

**FIGURE 2:**
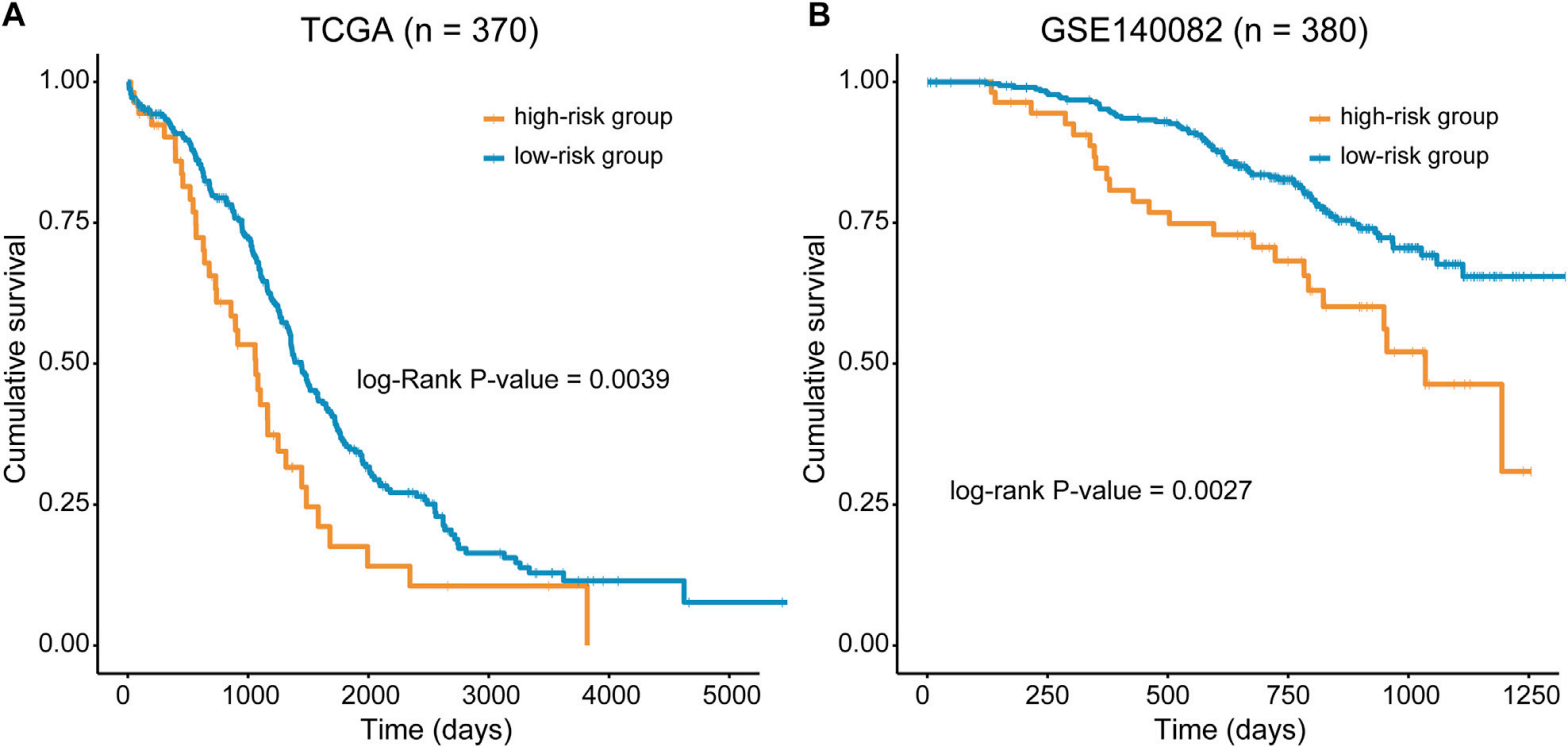
Kaplan–Meier survival curves of OS between the high- and low-risk groups stratified by the RMGS score in TCGA dataset **(A)** and GSE140082 **(B)**.

The predictive ability of RMGS was further validated in two large-scale independent validation datasets, including TCGA (n = 370) and GSE140082 (n = 380). With the RMGS model, the corresponding patients from TCGA dataset were also divided into two distinct risk groups with significantly different OS (*p*-value = 0.0039; see Figure 2A). In the univariate Cox proportional hazards regression analysis, the hazard ratio (HR) of a high RMGS score compared with a low RMGS score for OS was 1.684 in the TCGA dataset (*p*-value = 0.004; 95% confidence interval (CI), 1.578–2.409) (see Table 1), indicating that RMGS was significantly correlated with ovarian cancer OS. To evaluate the robustness and versatility, the predictive power of RMGS was further validated in an independent GEO dataset (GSE140082) (see Materials and Methods). As shown in Figure 2B, the patients in GSE140082 were stratified into the high-risk and low-risk groups, and the patients in the high-risk group had a significantly poor outcome compared with those in the low-risk group (*p*-value = 0.0027; log-rank test). In the univariate analysis, the RMGS score was also shown to be significantly associated with patient OS (HR = 2.020, 95% CI, 1.263–3.230, and *p*-value = 0.003) (see Table 1).

**TABLE 1:** Univariable and multivariable analysis results of the RMGS in TCGA and GEO validation sets.

	Univariable analysis			Multivariable analysis		
	HR	95% Cl	*p*-value	HR	95% Cl	*p*-value
TCGA						
RMGS	1.684	1.578–2.409	0.004	1.633	1.136–2.348	0.008
Grade	1.286	0.965–1.714	0.085	1.030	0.770–1.377	0.841
Stage	1.111	0.827–1.493	0.483	1.321	0.983–1.777	0.064
Age	1.019	1.007–1.031	0.001	1.020	1.007–1.032	0.001
GSE140082						
RMGS	2.020	1.263–3.230	0.003	1.889	1.181–3.025	0.008
Age	1.035	1.013–1.058	0.001	1.033	1.011–1.056	0.002

To further examine whether the RMGS is an independent prognostic factor, multivariate Cox regression analysis was conducted, including the RMGS score and other conventional clinical factors as covariables. The results from TCGA dataset showed that the RMGS (HR = 1.633, 95% CI 1.136–2.348, *p*-value = 0.008) is an independent prognostic factor for OS after adjusting the clinical characteristics. However, the stage factor (HR = 1.321, 95% CI 0.983–1.777, *p*-value = 0.064) does not show survival significance (see Table 1). In the GSE140082 dataset, the RMGS still maintained a significant correlation with OS in the multivariate analysis (HR = 1.889, 95% CI 1.181–3.025, *p*-value = 0.008). These results demonstrated that the RMGS is independent of other conventional clinical factors for OS prediction.

### Functional and Immune Infiltration Associations With the RMGS

**FIGURE 3:**
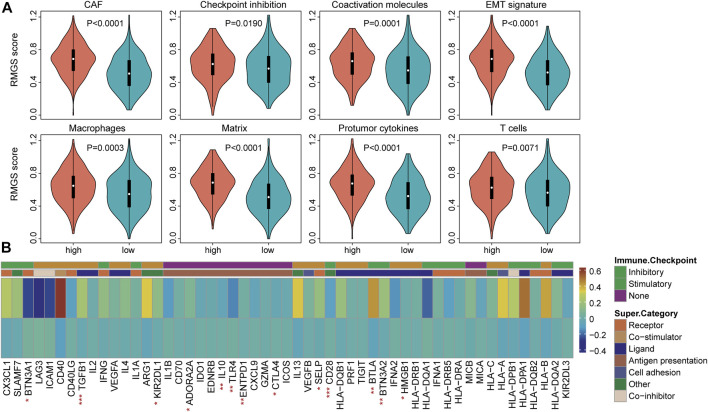
**(A)** Boxplots of the RMGS score between the high- and low-risk immune type groups **(B)** Comparison of the mean expression with expressions at the tumor sites relative to the high- and low-RMGS score in the immune checkpoint molecules. Statistical significance at the level of ∗ <0.05, ∗∗ <0.01, and ∗∗∗ <0.001.

To explore the functional implication of the RMGS signature, the correlation between the mRNA expression and RMGS score was computed, and the top 1% was selected as RMGS-related mRNAs. GO and Kyoto Encyclopedia of Genes and Genomes (KEGG) pathway enrichment analysis of these mRNAs demonstrated that the RMGS is highly associated with many key GO terms, including extracellular matrix organization, positive regulation of cytokine production, and positive regulation of cell adhesion. At the KEGG pathway aspects, the RMGS was associated with many tumor-related pathways, such as the ECM receptor interaction, cell adhesion molecules, cytokine–cytokine receptor interaction, and TNF signaling pathway (see Supplementary Figure S2). To test whether there exist associations between the RMGS score and immune infiltration level, we further utilized the single-sample GSEA method to calculate the infiltration score of 19 immune subpopulations based on corresponding marker genes (Subramanian et al., 2005). As shown in Figure 3A, some immune characterization displayed significant associations with the RMGS score. Also, the CAF, macrophages, and T-cell immune functions were significantly enriched in the high RMGS group. These results demonstrated that the RMGS signature is not only associated with patient prognosis but is also an indicator of the immune dysregulation condition in ovarian cancer.

We further investigated the correlation of intrinsic immune escape mechanisms with RMGS using TCGA dataset. Some potential factors that determine tumor immunogenicity were compared between the high- and low-RMGS groups. Leukocyte fraction, nonsilent mutation rate, and SNV neoantigens were significantly higher in the high RMGS group than in the low RMGS group (see Supplementary Figure S3). Another potentially important intrinsic immune escape mechanism is the expression of immune checkpoint molecules after immune stimulation. Figure 3B illustrated that the expression levels of some immune checkpoint molecules were significant between the high- and low-RMGS groups. This result indicated that tumor samples with a high RMGS score expressed immune checkpoint molecules to evade immune killing after immune stimulation, including TGFB1, Il10, CD28, BTLA, and BTN3A2.

### Involvement of RMGS in ovarian cancer molecular subtypes, immune subtypes, and therapeutic benefits

**FIGURE 4:**
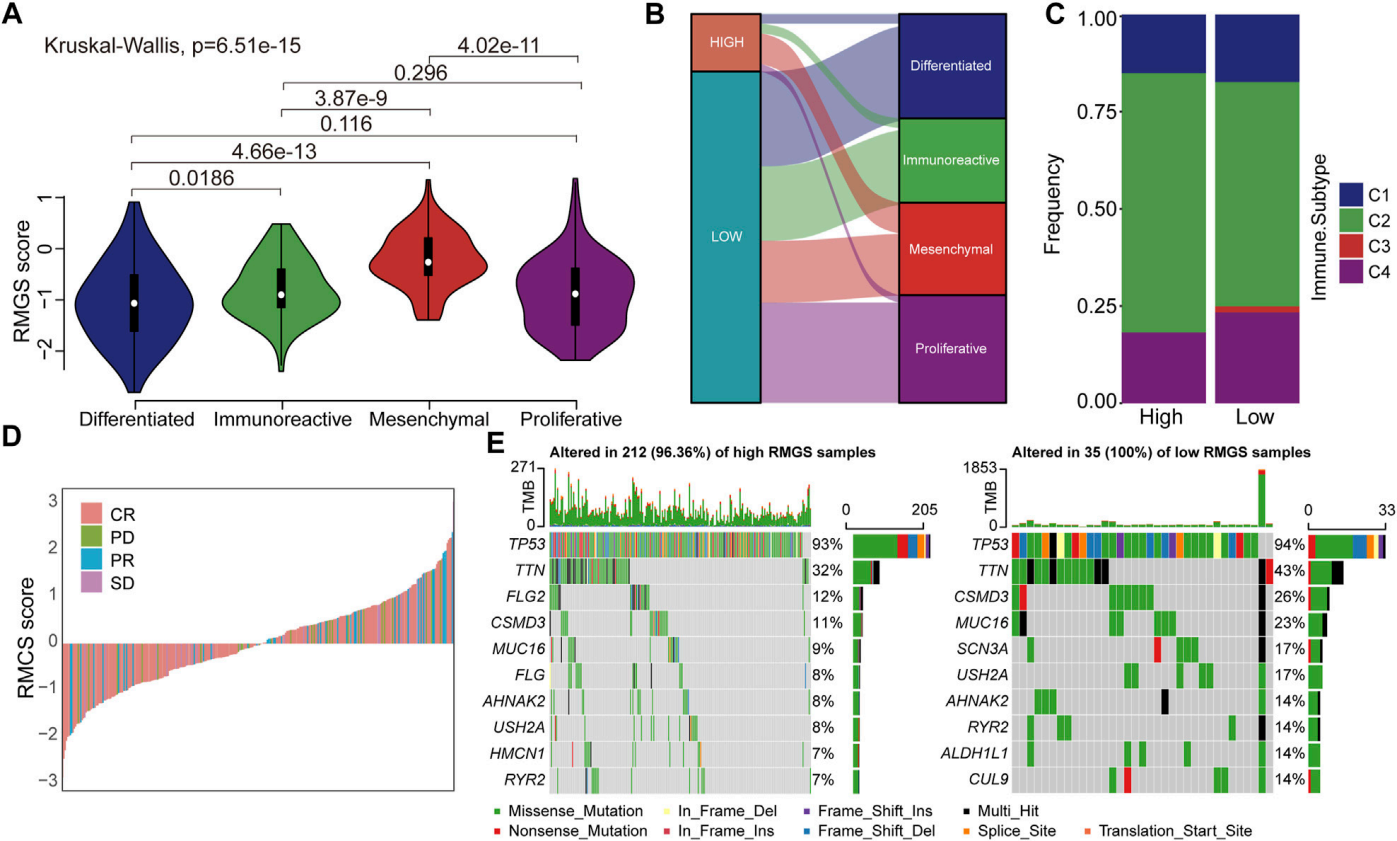
**(A)** Violin plots illustrated the correlation between the RMGS score and molecular subtypes **(B)** Alluvial diagram for the RMGS groups versus different intrinsic molecular subtypes **(C)** Association between the RMGS and the immune subtypes **(D)** Waterfall plot illustrated the RMGS score with different immunotherapy responses **(E)** Oncoplot of top 10 mutation genes in high- and low-RMGS groups.

We then investigated the difference and distribution of intrinsic molecular subtypes within two RMGS groups. As illustrated in Figure 4A, the mesenchymal subtype had a significantly higher RMGS score than other molecular subtypes. A significant difference was found among four molecular subtypes according to the Kruskal–Wallis test (*p*-value = 6.51E-15). For the TCGA dataset, an imbalance in terms of differentiated molecular subtype was noticed (Figure 4B). For the ovarian cancer immune subtypes, the samples with high RMGS scores displayed more C2 subtypes; however, the samples with low RMGS scores displayed the C3 subtype which was not included in another group (Figure 4C). By analyzing the available response after clinical treatment information based on samples from TCGA database, the association between the response results and RMGS groups was tested. The waterfall plots illustrated the correlation of the RMGS scores with clinical response status (Figure 4D). Furthermore, we observed these two RMGS groups shared many common genes of the top 10 mutation genes, such as *TP53*, *TTN*, and *MUC16*. Also, the specific genes within samples with high RMGS might be the drivers of the prognostic performance (Figure 4E).

## Discussion

In this study, we performed a bioinformatics analysis by integrating single-cell RNA sequencing and bulk expression profiles of ovarian cancer and found recurrence markers between recurrence samples and primary samples. The functions of these markers are enriched in epithelial cell differentiation, wound response, and regulation of cell growth. Then, we further constructed RMGS, a recurrence marker–based risk model, based on expression profiles of ovarian cancer in the merged database from GEO and validated the RMGS with TCGA dataset. RMGS served as an independent prognostic factor of OS in ovarian cancer patients when considering stage and grade factors. In addition, the RMGS score is closely related to the functional activities of macrophages, EMT signatures, and T cells, showing its relevance with immune infiltration. Finally, we observed the associations of RMGS scores with molecular subtypes and immune subtypes and specific drivers involved in high- and low-RMGS groups.

Among the RMGS genes, a total of seven genes have previously been revealed to be related to ovarian cancer formation and prognosis. It was found that *BIRC3* is one of the inhibitors of apoptosis proteins (IAPs). A lower expression level of *BIRC3* is associated with a better prognosis for ovarian cancer patients, and *BIRC3* knockdown in ovarian cancer cells can recover their sensitivity to cisplatin (Hu et al., 2019). *CDH2* (which encodes N-cadherin) and CDH6 (Cadherin 6) are significantly overexpressed in advanced ovarian cancer (Liu et al., 2020; Bartolomé et al., 2021). The *DDIT4* gene encodes a protein whose main action is to inhibit mTOR under stress conditions, whilst several *in vitro* studies indicated that its expression favors cancer progression (Zhang et al., 2021). Upregulating IGF2 could enhance the proliferation, migration, and invasion capacities of ovarian cancer cells (Gao et al., 2019). Mucin 16 (MUC16) is a glycoprotein that is highly expressed in ovarian cancer cells. For malignant ovarian cancer, the *MUC16* overexpression promoted cell proliferation, migration, and invasion *via* the PI3K/AKT signaling pathway (Crawford et al., 2019). *DIRAS3* is an imprinted tumor suppressor gene that encodes a 26 kD GTPase with homology to RAS that inhibits cancer cell proliferation and motility. Re-expression of DIRAS3 in ovarian cancer xenografts also induced dormancy and autophagy. DIRAS3 can bind to Beclin1 forming the autophagy initiation complex that triggers autophagosome formation (Sutton et al., 2019).

**FIGURE 5:**
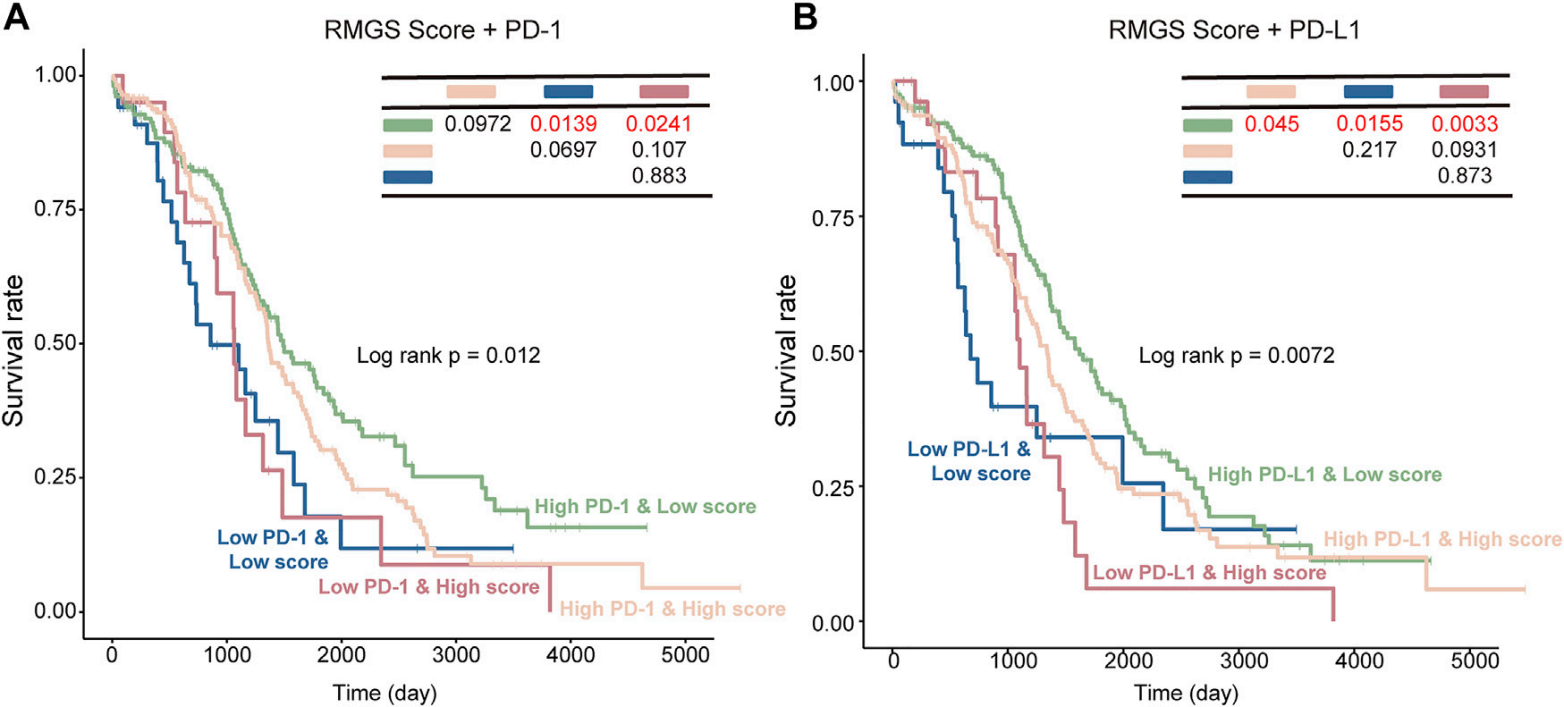
Kaplan–Meier survival curves of OS among the four patient groups stratified by the integration of the RMGS score and expression levels of PD-1 **(A)** and PD-L1 **(B)**.

To further test the association between RMGS scores and immune checkpoint, the effect of cross-talk between RMGS and two immune checkpoint genes (PD-1 and PD-L1) on patients’ survival was analyzed. First, TCGA patients were stratified into four groups based on the combination of RMGS and immune checkpoint genes, and then, a survival comparison was made among these four groups. The corresponding results revealed that the RMGS is able to distinguish the outcomes of patients with the same or similar levels of immune checkpoint genes (Figure 5). The patients with low RMGS and a high level of immune checkpoint genes tended to have significantly better survival prospects than the other three groups, whereas the patients with high RMGS and a low level of immune checkpoint genes tended toward the poorest outcome. The immune checkpoint inhibitors (ICIs) have opened a new era of cancer immunotherapy and provided a need for the identification of predictive biomarkers. The expression of immune checkpoint genes, including PD-1 and PD-L1, has been widely used as predictive biomarkers for ICI response (Duffy and Crown, 2019; Havel et al., 2019). PD-1 and PD-L1 have been reported to fulfill immuno-suppressive roles in tumor progression (Thibult et al., 2013; Ren et al., 2016). The interactions with immune checkpoint genes might reveal a candidate therapeutic predictive effect of RMGS on an ovarian cancer patient.

After adjusting for stage or grade factors, the RMGS remained an independent prognostic factor, which was capable of distinguishing worse versus improved survival outcomes in the validation set. In addition, it is the first model constructed by considering the epithelial cell markers differentially expressed between recurrence and primary samples and might reflect novel epithelial cell characterization involved in tumor recurrence. Compared with other single-cell RNA sequencing studies, one of the limitations is that the interaction of epithelial marker genes with other immune genes was not analyzed. For ovarian cancer treatment, the epithelial-mesenchymal status was involved in the patient’s response to cisplatin (Miow et al., 2015), and the difference between ovarian surface epithelial cells and fallopian tube secretory epithelial cells has been revealed (Auer et al., 2017). Therefore, the associations of our 13-gene signatures and epithelial functions need to be explored by experimental technology in further studies. Also, currently, although there exist some limitations, our study has provided a new understanding of epithelial cell markers in ovarian cancer patient prognosis and offered immunotherapy practice instructions for physicians.

## Data Availability

The original contributions presented in the study are included in the article/Supplementary Material; further inquiries can be directed to the corresponding author.
