# Supplementary figures and images for: Identification of a Recurrence Gene Signature for Ovarian Cancer Prognosis by Integrating Single-Cell RNA Sequencing and Bulk Expression Datasets
Source: Front Genet. 2022 Jun 8;13:823082. doi: 10.3389/fgene.2022.823082 (PMC9214038; doi:10.3389/fgene.2022.823082)

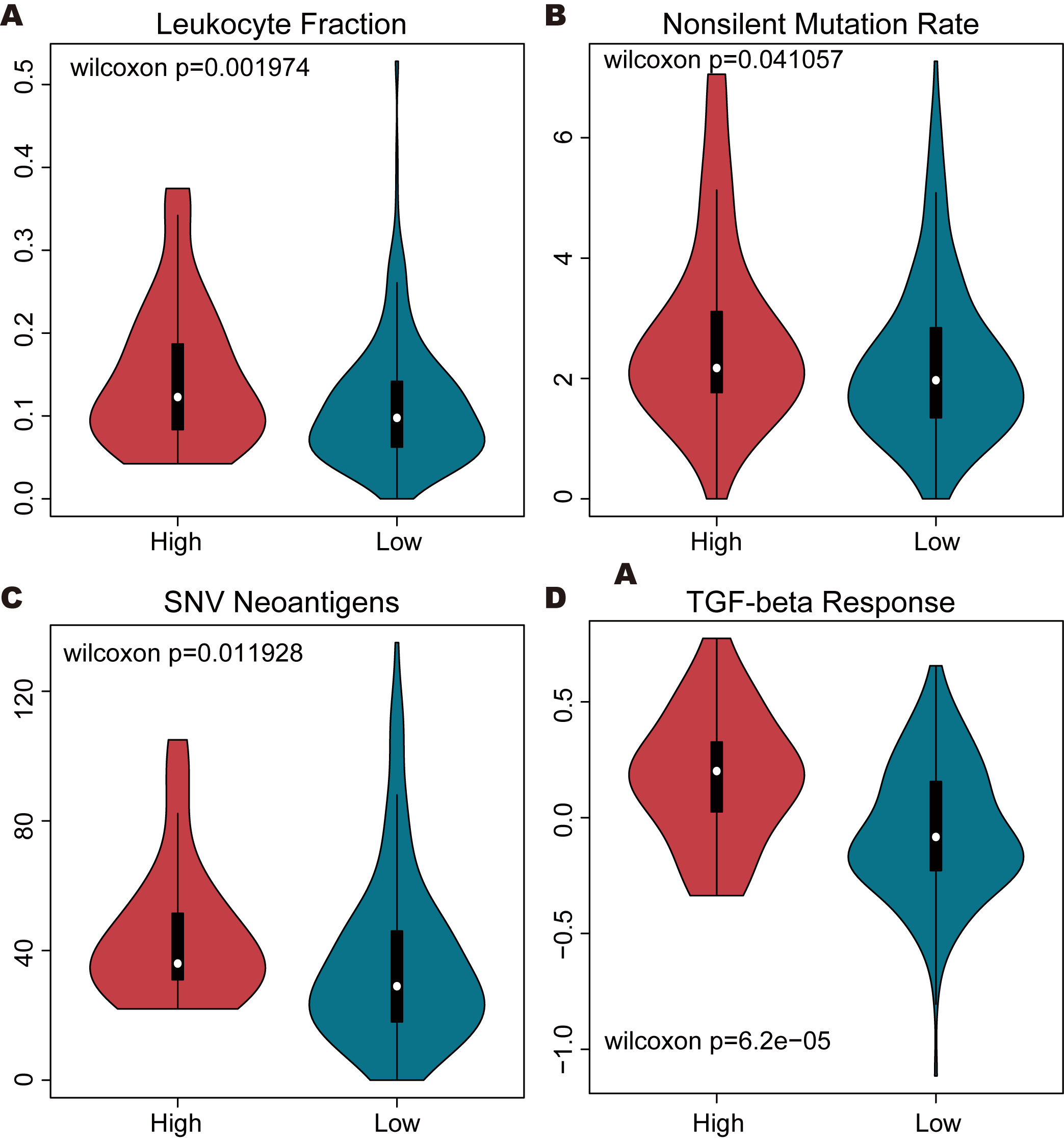

Supplement: Supplementary file 3 [file Image3.TIF]

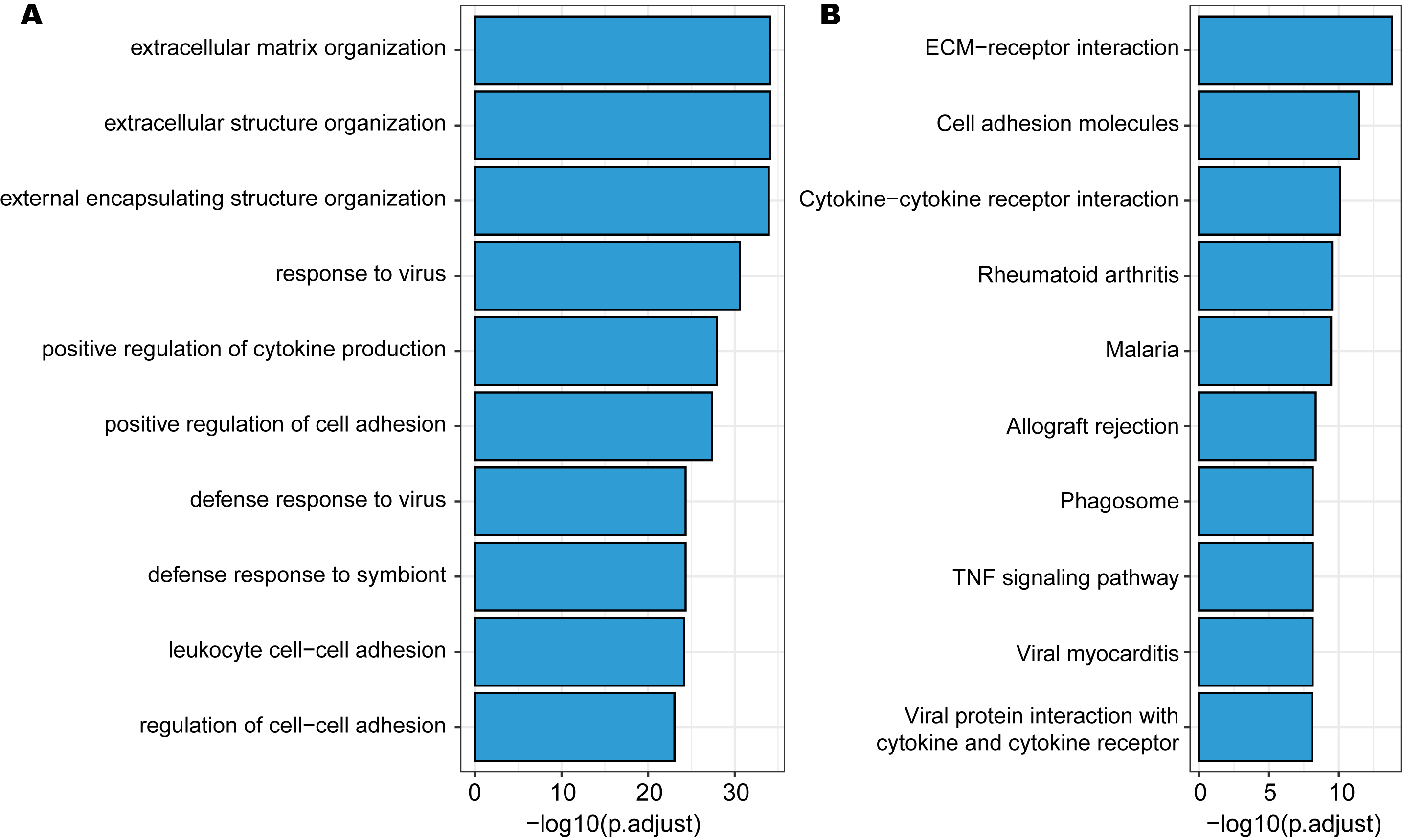

Supplement: Supplementary file 4 [file Image2.TIF]

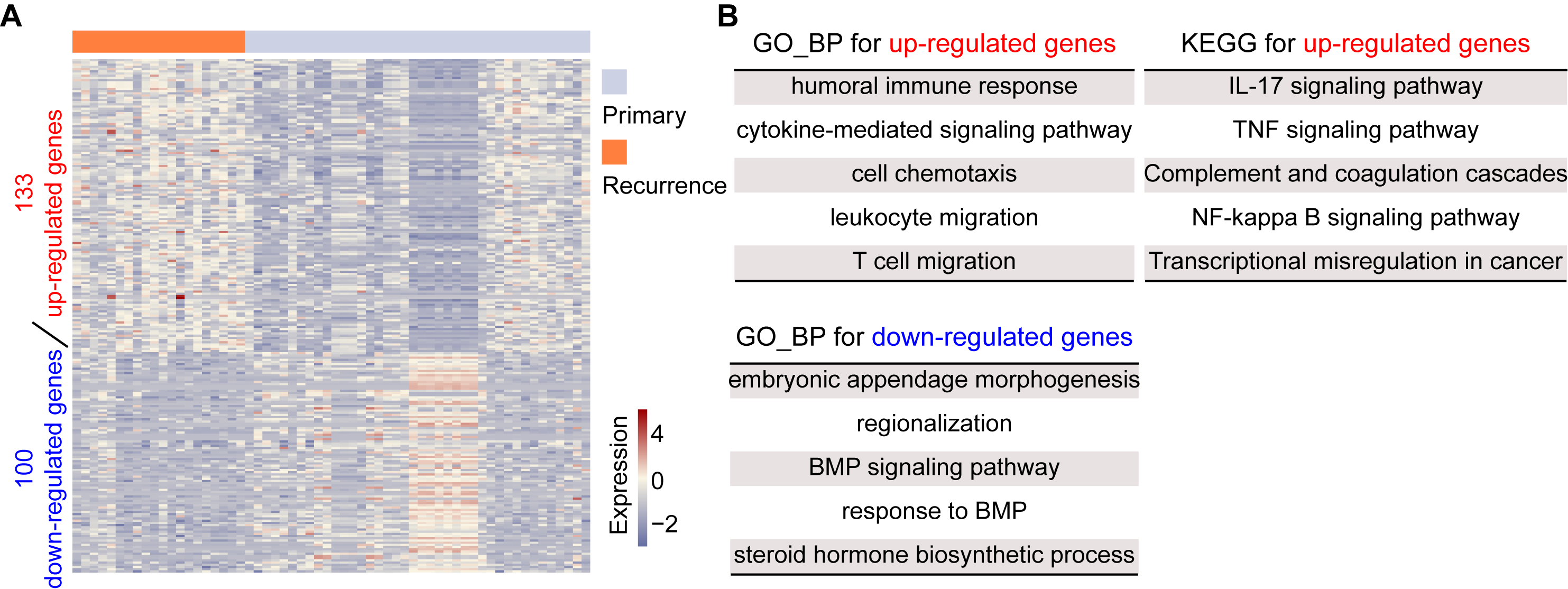

Supplement: Supplementary file 6 [file Image1.TIF]
